# Adherence to Electronic Health Tools Among Vulnerable Groups: Systematic Literature Review and Meta-Analysis

**DOI:** 10.2196/11613

**Published:** 2020-02-06

**Authors:** Jelena Arsenijevic, Lars Tummers, Niels Bosma

**Affiliations:** 1 Utrecht University School of Governance Faculty of Law Economics and Governance Utrecht Netherlands; 2 Utrecht University School of Economics Faculty of Law Economics and Governance Utrecht Netherlands

**Keywords:** eHealth, digital health, disparities in health care, meta-analysis

## Abstract

**Background:**

Electronic health (eHealth) tools are increasingly being applied in health care. They are expected to improve access to health care, quality of health care, and health outcomes. Although the advantages of using these tools in health care are well described, it is unknown to what extent eHealth tools are effective when used by vulnerable population groups, such as the elderly, people with low socioeconomic status, single parents, minorities, or immigrants.

**Objective:**

This study aimed to examine whether the design and implementation characteristics of eHealth tools contribute to better use of these tools among vulnerable groups.

**Methods:**

In this systematic review, we assessed the design and implementation characteristics of eHealth tools that are used by vulnerable groups. In the meta-analysis, we used the adherence rate as an effect size measure. The adherence rate is defined as the number of people who are repetitive users (ie, use the eHealth tool more than once). We also performed a meta-regression analysis to examine how different design and implementation characteristics influenced the adherence rate.

**Results:**

Currently, eHealth tools are continuously used by vulnerable groups but to a small extent. eHealth tools that use multimodal content (such as videos) and have the possibility for direct communication with providers show improved adherence among vulnerable groups.

**Conclusions:**

eHealth tools that use multimodal content and provide the possibility for direct communication with providers have a higher adherence among vulnerable groups. However, most of the eHealth tools are not embedded within the health care system. They are usually focused on specific problems, such as diabetes or obesity. Hence, they do not provide comprehensive services for patients. This limits the use of eHealth tools as a replacement for existing health care services.

## Introduction

### Background

Amra is a fictional 56-year-old Turkish migrant who has lived in Germany for more than 20 years. Although she recognized the first signs of menopause, she felt ashamed to visit her male general practitioner (GP) and talk about this. In addition, her German is not good. She discovered through her network of Turkish women that there is an app called Intelligent Health Assistant that can be downloaded on her mobile phone. This app can help her find information about menopause in both Turkish and German. Furthermore, the app allows her to make an appointment with a female doctor [1]. The above example shows how innovative communication technologies such as electronic health (eHealth) tools can be used to provide better information of, and access to, health care services for vulnerable groups such as migrants [1].

eHealth tools are increasingly being applied in health care [2]. They are known by different names, such as eHealth, informational communication technologies in health, consumer health information technologies, mobile health, Web-based health platforms, or telemedicine [3]. Usually, they are computer- and Web-based tools that are intended to improve quality of health care, health outcomes, access to health care services, and patients’ quality of life [4]. Examples of eHealth tools include patient portals, Web-based platforms that offer health care tools, or mobile phone apps. eHealth tools can use different technologies such as Web platforms developed for that purpose or social media platforms such as Facebook. Some of them are specifically developed for smartphones, whereas others can be used on any digital device [5]. Different services can be provided by eHealth tools—for example, making appointments, checking the results of laboratory tests, or participation in Web-based prevention programs. The first eHealth tools were developed in the United States. Today, many governments in Europe also advocate the use of eHealth tools within health care systems [6]. Different stakeholders are involved in their development. Some eHealth tools are developed in cooperation with health care providers. Others (also known as consumer eHealth) are developed by for-profit and nonprofit parties—small entrepreneurs or big companies—and are available on the open market [7].

It is asserted that eHealth tools have advantages compared with traditional delivery of health care services [8]. One of the potential advantages of using eHealth tools is that they can facilitate better patient-provider interactions. Of particular importance is the direct patient-provider interaction through eHealth tools that eliminates the need for physical appointments. It is assumed that such interactions can enhance the active participation of patients and lead to a more patient-centered care [9]. Furthermore, these tools mostly use encrypted Web platforms or apps that can capture personal data. This secures privacy for patients. In addition, with eHealth tools, users do not need to make an appointment to communicate with health care providers. Thus, users have quicker access to health care providers [10].

Although the advantages of using these tools in health care are well described, it is unknown to what extent these tools are effective when used by vulnerable groups. Vulnerable population groups are defined as social groups that have an increased risk for adverse health outcomes [11,12]. Vulnerable population groups include people with low socioeconomic status, older adults, single parents, minorities, or immigrants [5,13,14]. These groups tend to have lower health outcomes and experience more difficulties in accessing health care services compared with the general population [15]. Most of these difficulties are related to social injustice and can be improved by efficient health policies or by adopting innovative health tools such as eHealth tools [12]. Previous studies have shown that the use of eHealth tools among vulnerable groups can have double-folded effects [16]. In some cases, eHealth tools improve access to health care. In our fictional example of Amra, it helped her and catered to her current needs. In her case, the mobile phone app provided improved access to adequate information and health care services. However, innovative tools do not always have positive effects among vulnerable groups. In some cases, these tools can increase the disparities that exist between vulnerable groups and the general population [17]. For example, older adults who are not familiar with internet technology may not be able to make appointments via an electronic patient portal [18]. eHealth tools may then reduce access to health care for these groups.

However, information about the effectiveness of eHealth tools among vulnerable groups is still inconsistent. Previous studies have shown that effectiveness of eHealth tools among vulnerable groups is influenced by the level of adherence [19,20]. The term adherence was initially used for medication, but it is also used in other health areas [21]. Adherence is defined by the World Health Organization (WHO) as the “extent to which a person’s behavior—taking medication, following a diet, and/or executing lifestyle changes-corresponds with agreed recommendations from a health care provider” [22]. In the case of eHealth tools, there are many challenges in applying this WHO definition [21-24]. In 2005, Eysenbach was the first to notice that although in the case of medication adherence we often know what optimal dosage is, this is not always the case for eHealth tools [21,25]. Some authors have proposed the concept of *intended use* or *use as it is designed* [22,23]. However, this provides no justification for the level of intended use. Others argue that the use of all components of eHealth tools by all population groups might not be necessary. Some groups might achieve their personal goals by using only a few components [24]. Furthermore, different eHealth tools might require different intended uses to be effective in changing health outcomes [24,26]. For example, to change their lifestyle, users might be engaged with eHealth tools once per day for extended periods, whereas to maintain good self-management of chronic diseases, users need to be engaged several times per day [27,28]. This means that adherence can be influenced by users’ characteristics as well as the characteristics of the goal of the eHealth tool. On this basis, different metrics of adherence are proposed—some authors propose measures such as the number of log-ins or the number of characters that are typed every time a person is logged in or the number of Web pages accessed [22]. Others propose the use of different measures such as the attrition rate or the dropout rate.

In previous studies, adherence to eHealth tools was compared among different population groups, including vulnerable groups [18,29,30]. Some of the studies report this percentage at the end of the intervention period, without reporting dropout rates across population groups. Not surprisingly, most of these studies concluded that the percentage of users from a vulnerable population is lower than that among the general population [31,32]. However, this does not imply that vulnerable groups did not achieve the intended use.

In this study, we used the method proposed by Sieverink et al [23] as operationalization category C level—“Assigned when the intended use of the technology was provided and justified using theory, evidence, or rationale.” We examined the number of repeated users for the eHealth tool after a period of time that is justified to be relevant for this eHealth tool.

Another drawback of the previous studies that assessed failure in use of eHealth tools among vulnerable groups was that the focus was typically limited to generic characteristics such as low health literacy, low education levels, and lack of access to fast internet [3,33,34]. However, the design and implementation characteristics of eHealth tools can also play a role in their effective use among vulnerable groups [8]. Previous findings have shown that design characteristics such as the type of technology used (mobile app or Web-based platform), use of multimodal content (use of videos, games, or quizzes), or the possibility of direct interaction between patients and providers can increase the use of eHealth tools among vulnerable groups [3]. Different vulnerable groups have different preferences regarding the type of technology used. Some vulnerable groups such as migrants or low-income single mothers prefer the use of mobile phones, whereas others such as chronically sick or older patients seem to prefer Web-based platforms [3,35,36]. Multimodal content facilitates the use of eHealth tools for vulnerable groups that have problems with health literacy (ability to understand, proceed, and make decisions with health information). Videos or games are less language saturated and can be understood and used by people with low health literacy [37]. To overcome the problem of a digital divide (lack of knowledge on how to use the internet) [38] and/or health literacy [39], eHealth tools sometimes use direct interaction between patients and providers. Direct interaction makes personalized information available to the patients, which consequently leads to a better understanding in patients [40]. On the basis of previous literature, we have also identified implementation characteristics that can lead to improved use of eHealth tools among vulnerable groups. One of these characteristics is the possibility to let eHealth tools be used by vulnerable groups exclusively or to introduce eHealth tools that are developed for the general population but can be easily adopted by vulnerable groups. The possibility of training related to the use of eHealth tools is also important for vulnerable groups. Reluctance to use eHealth tools may stem from feelings of incompetence in vulnerable groups. Training can help them overcome this problem [13].

### Objectives

On the basis of the above-mentioned information, this study had two goals. First, we aimed to identify the level of adherence toward eHealth tools among vulnerable groups. To this end, we conducted a systematic literature review and meta-analysis. Second, we aimed to establish how different design and implementation characteristics influence the level of adherence. To this end, we conducted a meta-regression. Identifying potentially successful designs for eHealth tools can help include these tools as a regular part of health care service delivery. Furthermore, if eHealth tools are adopted by vulnerable groups, they could improve access to health care services and even replace some of the existing services [41].

## Methods

### Reporting Standards

A systematic literature review was conducted in accordance with the Preferred Reporting Items for Systematic Reviews and Meta-Analysis (PRISMA) strategy [42]. In addition, we used PRISMA recommendations for a replicable meta-analysis (see Multimedia Appendix 1).

### Inclusion Criteria

We included studies that (1) examined the use of Web-based innovative technologies among at least one vulnerable group (older adults, chronically sick, minorities, people with low socioeconomic status, and migrants), (2) were published in peer-reviewed journals in English after 2007, (3) focused on people aged 18 years or older, and (4) reported the level of patient/user participation (adherence). Regarding the design, we included studies with the following designs: randomized controlled trial (RCT and similar designs such as pragmatic RCT), prospective longitudinal studies, pre- and postdesign studies, and cohort studies.

### Exclusion Criteria

We excluded studies with a qualitative research design, case studies, opinion papers, literature reviews or theoretical views, studies that assessed the use of Web-based technologies to address the education or decision-making process among medical providers, studies that examined new medical devices and their technical characteristics based on Web-based apps, studies that assessed psychometric instruments that are used to evaluate Web-based apps, and studies that evaluated Web-based population surveys.

### Search Strategy, Study Selection, and Data Extraction

First, we conducted an electronic search in the following databases: PubMed, Web of Knowledge, EBSCO, and CINAHL. All databases were searched from January 5, 2017, to January 5, 2018. To develop the search strategies, we checked two main sets of keywords: (1) eHealth tools and corresponding synonyms (Web-based information technologies, social media, internet based, electronic-records, Facebook, etc) and (2) health disparities and corresponding synonyms (disparity in health, vulnerable groups, or inequity). For both sets of keywords, we also checked the thesaurus and Medical Subject Headings terms. Second, we developed a search strategy for each database. The detailed strategy for PubMed is presented in Textbox 1. After the initial selection of studies, we checked their reference lists for additional literature. A publication from the reference list (bibliography) was included in the review after applying the same inclusion and exclusion criteria. Third, we conducted a forward search by looking up the studies that cited the included studies. For this purpose, we used PubMed. Fourth, we used literature review studies to check whether we included studies that have been identified in previous literature reviews. We used a PRISMA flowchart to present the search strategy.

**Search string used for PubMed.**

String used for PubMed: ((((((((((e-Health[Title/Abstract] OR eHealth[Title/Abstract]) OR ((“health”[MeSH Terms] OR “health”[All Fields]) AND (“Information (Basel)”[Journal] OR “information”[All Fields]) AND technologies[Title/Abstract])) OR patient portals[Title/Abstract]) OR telemedicine[Title/Abstract]) OR “social media”[MeSH Terms]) OR Facebook[Title/Abstract]) OR Twitter[Title/Abstract]) OR Web 2.0[Title/Abstract]) OR “internet”[MeSH Terms]) AND ((“health”[MeSH Terms] OR “health”[All Fields]) AND disparities[All Fields])) OR vulnerable [All Fields] OR disadvantaged [All Fields] and migrants [MESH]OR immigrants [MESH] OR low income [Title/Abstract] OR older adults [Title/Abstract]))

### Study Selection and Characteristics of the Selected Studies

**Figure 1:**
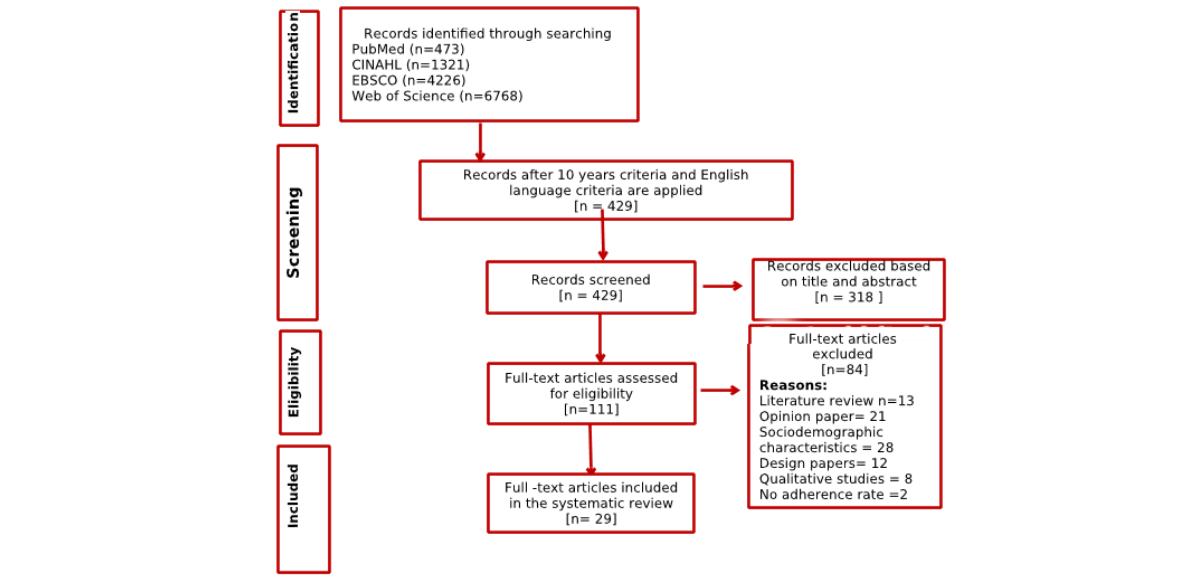
Searching strategy for PubMed I.

On the basis of the search strategy, we identified 473 publications. We presented the selection process through a PRISMA flowchart (Figure 1). After applying filters for English language and duration from 2007 to 2017, we were left with 429 publications. In the next step, we checked the titles and abstracts, resulting in 318 excluded studies (mostly studies addressing telemedicine, using providers as participants, or using data on an organizational level). Thereafter, we screened the remaining 111 publications. Among them were 13 literature reviews [4,34,36,43-51] and 21 opinion papers [6-9,13-15,52-65]. These were all excluded. We also excluded 28 studies that examined only sociodemographic characteristics of eHealth users and 12 studies that were design papers. In addition, we excluded eight studies because they were qualitative studies that used focus group methods to gather data. In total, we included 27 studies based on our inclusion and exclusion criteria [3,33,35,66-89].

For conducting search in the other three databases, we used combinations of all two keywords. The articles that were found within the other databases and met our inclusion and exclusion criteria were the same as those already identified with PubMed.

The summarized description of all selected articles is presented in Table 1. The detailed description of all included articles is presented in Multimedia Appendix 2.

**Table 1:** Summary of the study characteristics (N=27).

Study characteristics		Value	Study
**Year of publication, n (%)**			
	200	1 (4)	Kim et al [66]
	2010	2 (7)	Sarkar et al [67], Kerr et al [68]
	2011	2 (7)	Ancker et al [33], Goel et al [69]
	2013	5 (19)	Ronda et al [35], Nazi et al [81], Osborn et al [70], Joseph et al [72], Ryan et al [88]
	2014	2 (7)	Steinberg et al [86], Herring et al [75]
	2015	6 (22)	Campbell et al [74], Foster et al [3], Billings et al [76], Smith et al [77], Levy et al [78], Jhamb et al [79]
	2016	5 (19)	Joseph et al [73], Gordon and Hornbrook [80], Aalbers et al [84], Cavallo et al [85], Bickmore et al [87]
	2017	4 (15)	Cullen et al [71], Ernsting et al [82], Arcury et al [83], Buis et al [89]
**Country, n (%)**			
	United States	23 (85)	Kim et al [66], Sarkar et al [67], Ancker et al [33], Goel et al [69], Osborn et al [70], Cullen et al [71], Joseph et al [72], Joseph et al [73], Campbell et al [74], Herring et al [75], Billings et al [76], Smith et al [77], Levy et al [78], Jhamb et al [79], Gordon and Hornbrook [80], Nazi et al [81], Foster et al [3], Arcury et al [83], Cavallo et al [85], Steinberg et al [86], Bickmore et al [87], Ryan et al [88], Buis et al [89]
	Other	4 (15)	Kerr et al [68], Ronda et al [35], Ernsting et al [82], Aalbers et al [84]
**Design of the study, n (%)**			
	Cohort study	7 (26)	Kim et al [66], Sarkar et al [67], Kerr et al [68], Smith et al [77], Jhamb et al [79], Gordon and Hornbrook [80], Nazi et al [81]
	Randomized controlled trial	12 (44)	Ronda et al [35], Osborn et al [70], Cullen et al [71], Joseph et al [73], Herring et al [75], Billings et al [76], Levy et al [78], Ernsting et al [82], Steinberg et al [86], Bickmore et al [87], Ryan et al [88], Buis et al [89]
	One group pre- to postdesign	5 (19)	Joseph et al [72], Campbell et al [74], Foster et al [3], Aalbers et al [84], Cavallo et al [85]
	Longitudinal studies	3 (11)	Ancker et al [33], Goel et al [69], Arcury et al [83]
**Sample size, n (%)**			
	N>100	17 (62)	Kim et al [66], Sarkar et al [67], Kerr et al [68], Ancker et al [33], Goel et al [69], Ronda et al [35], Cullen et al [71], Smith et al [77], Jhamb et al [79], Gordon and Hornbrook [80], Nazi et al [81], Ernsting et al [82], Arcury et al [83], Aalbers et al [84], Cavallo et al [85], Steinberg et al [86], Buis et al [89]
	N<100	10 (37)	Osborn et al [70], Joseph et al [72], Joseph et al [73], Campbell et al [74], Herring et al [75], Billings et al [76], Levy et al [78], Foster et al [3], Bickmore et al [87], Ryan et al [88]
**Area of health care where electronic health tool is applied, n (%)**			
	Primary care	5 (18.5)	Ancker et al [33], Nazi et al [81], Ernsting et al [82], Arcury et al [83], Bickmore et al [87]
	Diabetes	4 (14.8)	Sarkar et al [67], Ronda et al [35], Levy et al [78], Ryan et al [88]
	Cardiovascular diseases	2 (7.4)	Kerr et al [68], Buis et al [89]
	Obesity	8 (29.6)	Kim et al [66], Cullen et al [71], Joseph et al [72], Joseph et al [73], Herring et al [75], Aalbers et al [84], Cavallo et al [85], Steinberg et al [86]
	Other chronic diseases	4 (14.8)	Osborn et al [70], Campbell et al [74], Smith et al [77], Jhamb et al [79]
	Reproductive health	2 (7.4)	Goel et al [69], Gordon and Hornbrook [80]
	Nursing home	2 (7.4)	Billings et al [76], Foster et al [3]
**Target population, n (%)**			
	Minorities	12 (44.4)	Kim et al [66], Cullen et al [71], Joseph et al [72], Joseph et al [73], Campbell et al [74], Billings et al [76], Foster et al [3], Arcury et al [83], Steinberg et al [86], Bickmore et al [87], Ryan et al [88], Buis et al [89]
	Low-income people	5 (18.5)	Ancker et al [33], Herring et al [75], Levy et al [78], Ernsting et al [82], Cavallo et al [85]
	Older adults	4 (14.8)	Goel et al [69], Smith et al [77], Gordon and Hornbrook [80], Aalbers et al [84]
	Chronically sick	6 (22.5)	Sarkar et al [67], Kerr et al [68], Ronda et al [35], Osborn et al [70], Jhamb et al [79], Nazi et al [81]
Quality score of the studies, mean (SD)		21.07 (2.90); minimum: 17.00, maximum: 31.00	All

### Quality Assessment

To assess the quality of the included studies, we used the quality assessment proposed by Zingg et al [90]. This tool is known as Integrated quality Criteria for the Review of Multiple Study designs (ICROMS). ICROMS allows us to calculate the quality scores for articles with different study designs such as RCTs, cohort studies, or controlled before-and-after studies. It consists of a clear and transparent scoring system accompanied by a decision matrix for each of the indicators that is related to the quality of the article. Each indicator gets a score of 2 if the criteria for the indicator are met, 0 if this is not the case, and 1 if it is unknown whether the criteria were met. In total, 33 indicators are grouped in seven dimensions, namely, clear aims and justification, managing bias in sampling or between groups, managing bias in outcome measurements and blinding, managing bias in follow-up, managing bias in other study aspects, analytical rigor, and managing bias in reporting/ethical considerations. The score depends on the design of the study.

### Data Extraction and Outcome Measures

In accordance with the PRISMA guidelines, we extracted the following characteristics for each study: year of publication, country of origin, study design, target population, area of health care where eHealth tool is applied, and quality of the study. To calculate the adherence level, we also extracted the total sample size (N), the sample size for those who used eHealth more than once (n2), and the sample size for those who are registered but did not use eHealth tools more than once—uptake (n1). We also calculated the probability of continuous users (intended adherence; *P*2=n2/N) and probability of one-time users (*P*1=n1/N). These data are presented in Multimedia Appendix 2.

We also extracted the following design characteristics: the possibility to have direct contact with a medical provider, use of multimodal content (videos, games, and quizzes), and the type of technology used (patient portal, Web-based portal, or mobile app). Next, we extracted the following implementation characteristics: target group addressed by eHealth tools, whether the eHealth tool is exclusive for the target group or can be used among the whole population (inclusive), and the possibility of training. These data are presented in Multimedia Appendix 2. All data were extracted by 1 researcher.

### Data Synthesis and Analysis

To assess the adherence among vulnerable population groups, we conducted a random-effects meta-analysis. We calculated the ratio between the probability of nonusers (people who did not use eHealth tools or those who used eHealth tools once, usually during registration) and the probability of continuous users (intended adherence). People who used eHealth tools only once, when they were registered, are similar to nonusers. They might be registered by their health care providers or family members, but they had never activated and used their account. We calculated the probability of nonusers as *P*1=n1/N, where N is the total sample, and n1 is the number of people who used eHealth tools only once or did not use it at all.

**Figure 2:**
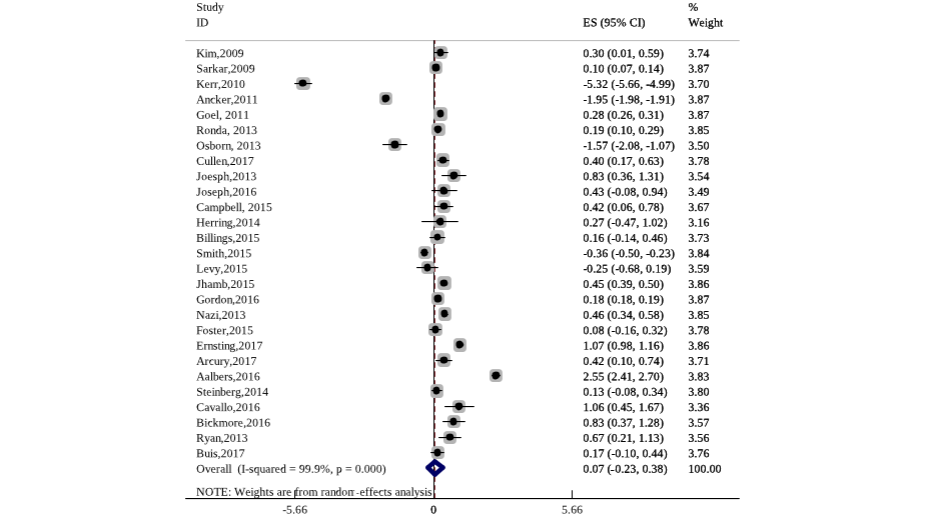
Results from meta-analysis-effect size adherence rate.

If the study reported the number of nonusers, we compared the repetitive users with nonusers. Next, we calculated the probability of continuous users as *P*2=n2/N, where n2 is the number of users who used eHealth tools as it was designed and in a way that was justified to be relevant for this eHealth tool. Thereafter, we calculated the estimate of the effect size measure—risk ratio (RR=*P*2/*P*1) and made the logarithm transformation log(RR). Logarithm transformation was usually used when the included studies had a different research design [91]. For the visual representation of the results, we used a funnel plot (see Figure 2). Between studies, heterogeneity was assessed through the I^2^ statistic (with a value higher than 75% considered as large).

We performed a meta-regression to assess the extent to which different design and implementation characteristics influence the adherence rate among vulnerable population groups. In the meta-regression, the dependent variable was the size of the effect estimates from the individual studies. As explanatory variables, we included design and implementation characteristics such as the type of technology used for the eHealth tool (patient portal, Web-based tool, or mobile app), the presence of multimodal content (yes/no), the availability of training for the use of eHealth tools, and direct interaction with a medical doctor (yes/no). The quality of the study was used as a covariate. The results of the meta-analysis (effect size measures for adherence) might be saturated with different sampling methods and different study designs. This can lead to heterogeneity in effect size measures. Meta-regression can also help explore the reasons for heterogeneity. In the meta-regression output, heterogeneity between the studies was measured through the I^2^ statistic (with a value higher than 75% considered as large). The proportion of between-study variance explained by the model was calculated through tau squared. For both meta-analysis and meta-regression, we extracted data from 27 studies.

### Publication Bias Tests

It has been shown that studies that report statistically significant results or clinically relevant results are published more often [92]. This can lead to publication bias—that is, effect sizes of studies included in the meta-analysis differ from the general effect size when considering all studies [93]. To test for publication bias related to standardized adherence, we applied the Begg and Mazumdar rank correlation tests and the Egger test. The results from the publication bias test are presented in the Results section.

## Results

### Main Study Characteristics

We included 27 studies. Table 1 presents the characteristics of the included studies. Most studies were from the United States (23/27, 85%) and were published in the period 2013 to 2017 (22/27, 82%). In addition, most studies had an RCT design (12/27, 44%). However, it is also worth mentioning that most RCTs were derived from larger cohort studies. This means that randomization has been conducted between registered and repeated users. Furthermore, most studies were related to primary care or health promotion (eg, addressing the problem of obesity). The studies related to primary care were mostly associated with patient portals, such as kp.org portals from different states in the United States, the MyChart portal from the United Kingdom, or My Health at Vanderbilt (also in the United States), that aimed to provide better access to primary care for chronically sick users. eHealth tools that address the problem of obesity were usually Web portals. They presented extensions of already existing health promotion interventions: these interventions were not delivered in community centers; these were delivered through Web-based portals. This was, for instance, the case with the Muévete Alabama study that aimed to decrease obesity among Latinas in the United States [94]. Our results showed that most studies targeted minorities (12/27, 44%). In addition, more than half of the studies used a sample size of more than 100. The mean value of quality score was 21. This can be described as a middle-quality score. Most studies had the lowest score on the dimension managing bias in sampling or between groups. Our results also showed that some design characteristics, such as type of technology, were related to some characteristics of the included studies.

### Design and Implementation Characteristics of Electronic Health Tools

In Table 2, we summarize the design and implementation characteristics of the eHealth tools that are used by vulnerable population groups.

The number of studies related to patient portals and Web-based platforms was quite high (22/27, 82%), whereas there were fewer studies that evaluated mobile apps (5/27, 19%). Our results also showed that almost all eHealth tools (23/27, 86%) provided the possibility for direct communication with the provider. Conversely, the number of eHealth tools that used multimodal content was small (10/27, 37%). The studies that used multimodal content were usually Web-based portals that provide videos or games. One example is a Dutch study that aimed to improve the lifestyle of older adults [78]. Among the implementation characteristics, the possibility of training for the use of the eHealth tool was rare—only 5 (5/27, 19%) studies reported it. The number of eHealth tools exclusively made for vulnerable groups was similar to the number of tools that can be applied to a general population (13/27, 48% vs 14/27, 52%).

Our results also showed that some design characteristics, such as the type of technology, were related to the design. Most studies with an RCT design were related to the use of Web-based platforms, whereas those with a cohort design were related to the use of patient portals. Patient portals were related to primary care services or nursing homes, whereas Web-based platforms were mostly related to the problems of obesity. Table 3 presents these results.

**Table 2:** Design and implementation characteristics (N=27).

Design and implementation characteristics			Value, n (%)		Study	
**Design characteristics**						
	**Type of technology used**					
		Web-based platforms		12 (44)		Kerr et al [68], Cullen et al [71], Joseph et al [72], Joseph et al [73], Campbell et al [74], Billings et al [76], Arcury et al [83], Aalbers et al [84], Cavallo et al [85], Steinberg et al [86], Bickmore et al [87], Ryan et al [88]
		Patient portals		10 (37)		Kim et al [66], Sarkar et al [67], Ancker et al [33], Goel et al [69], Ronda et al [35], Osborn et al [70], Smith et al [77], Jhamb et al [79], Gordon and Hornbrook [80], Nazi et al [81]
		Mobile app		5 (19)		Herring et al [75], Levy et al [78], Foster et al [3], Ernsting et al [82], Buis et al [89]
	**Use of multimodal content (yes=1; no=0)**					
		Yes		10 (37)		Cullen et al [71], Joseph et al [72], Joseph et al [73], Campbell et al [74], Billings et al [76], Ernsting et al [82], Aalbers et al [84], Cavallo et al [85], Steinberg et al [86], Bickmore et al [87]
		No		17 (63)		Kim et al [66], Sarkar et al [67], Kerr et al [68], Ancker et al [33], Goel et al [69], Ronda et al [35], Osborn et al [70], Herring et al [75], Smith et al [77], Levy et al [78], Jhamb et al [79], Gordon and Hornbrook [80], Nazi et al [81], Foster et al [3], Arcury et al 112], Ryan et al [88], Buis et al [89]
	**Possibility of direct interaction with provider (yes=1; no=0)**					
		Yes		23 (86)		Kim et al [66], Sarkar et al [67], Kerr et al [68], Ancker et al [33], Goel et al [69], Ronda et al [35], Osborn et al [70], Cullen et al [71], Joseph et al [72], Joseph et al [73], Herring et al [75], Smith et al [77], Levy et al [78], Jhamb et al [79], Gordon and Hornbrook [80], Nazi et al [81], Foster et al [3], Ernsting et al [82], Arcury et al [83], Aalbers et al [84], Cavallo et al [85], Bickmore et al [87], Ryan et al [88], Buis et al [89]
		No		4 (15)		Campbell et al [74], Billings et al [76], Steinberg et al [86], Ernsting et al [82]
**Implementation characteristics**						
	**Type of target group**					
		Minorities		12 (44)		Kim et al [66], Cullen et al [71], Joseph et al [72], Joseph et al [73], Campbell et al [74], Billings et al [76], Foster et al [3], Arcury et al [83], Steinberg et al [86], Bickmore et al [87], Ryan et al [88], Buis et al [89]
		Low-income people		5 (19)		Ancker et al [33], Herring et al [75], Levy et al [78], Ernsting et al [82], Cavallo et al [85]
		Older adults		4 (15)		Goel et al [69], Smith et al [77], Gordon and Hornbrook [80], Aalbers et al [84]
		Chronically sick		6 (23)		Sarkar et al [67], Kerr et al [68], Ronda et al [35], Osborn et al [70], Jhamb et al [79], Nazi et al [81]
	**Exclusive or inclusive for target group**					
		Exclusive		14 (52)		Kim et al [66], Sarkar et al [67], Ronda et al [35], Cullen et al [71], Joseph et al [73], Herring et al [75], Levy et al [78], Nazi et al [81], Foster et al [3], Aalbers et al [84], Steinberg et al [86], Bickmore et al [87], Ryan et al [88], Buis et al [89]
		Inclusive		13 (48)		Kerr et al [68], Ancker et al [33], Goel et al [69], Osborn et al [70], Joseph et al [72], Campbell et al [74], Billings et al [76], Smith et al [77], Jhamb et al [79], Gordon and Hornbrook [80], Ernsting et al [82], Arcury et al [83], Cavallo et al [85]
	**Possibility of training**					
		Yes		5 (19)		Kim et al [66], Sarkar et al [67], Kerr et al [68], Joseph et al [72], Bickmore et al [87]
		No		22 (82)		Ancker et al [33], Goel et al [69], Ronda et al [35], Osborn et al [70], Cullen et al [71], Joseph et al, 2016. [73], Campbell et al [74], Herring et al [75], Billings et al [76], Smith et al [77], Levy et al [78], Jhamb et al [79], Gordon and Hornbrook [80], Nazi et al [81], Foster et al [3], Ernsting et al [82], Arcury et al [83], Aalbers et al [84], Cavallo et al [85], Steinberg et al [86], Ryan et al [88], Buis et al [89]

**Table 3:** Type of technology used and study characteristics (N=27).

Type of technology used	Study designs, n (%)	Area of health care where electronic health tool is applied, n (%)	Target population, n (%)
Web-based platform (n=11)	RCT^a^, 6 (22); others, 5 (19)	Obesity, 6 (22); others, 5 (19)	Minorities, 8 (30); others 3 (11)
Patient portals (n=11)	Cohort, 6 (22); others, 5 (19)	General practice, 4 (15); others, 7 (26)	Chronically sick, 5 (19); elderly, 3 (11); minorities, 2 (8); low-income, 1 (4)
Mobile apps (n=5)	RCT, 3 (11); others, 2 (7)	—^b^	Low-income people, 3 (11); others, 2 (7)

^a^RCT: randomized controlled trial.

^b^Missing data.

### Adherence to Electronic Health Tools Among Vulnerable Groups—Results From Meta-Analysis

To examine the extent to which vulnerable population groups adopted eHealth tools, we conducted meta-analyses. Results from the meta-analysis on adherence effect size measures showed that the difference in proportion between intended adherers and only registered users was 7% (95% CI −0.23 to 0.38). They showed that users from vulnerable groups adopted eHealth tools for continuous use. However, the difference between registered and repetitive users was still small. In Figure 2, the middle value on the axis should be 0.5 instead of the standard—0. The reason was that we examined the difference in users who registered once but not in continuous users and repetitive users. This means that all users had a chance to potentially use the eHealth tool. I^2^ tests show high between-study heterogeneity.

### Design and Implementation Characteristics of Electronic Health Tools and Adherence

To examine how different design and implementation characteristics influence the adherence rate, we applied meta-regression. The results from meta-regression (Table 4) showed that studies that evaluated eHealth tools with multimodal content and direct patient-provider interaction reported a higher adherence rate. This means that the use of multimodal content and the possibility of having direct contact with providers seem to increase the adoption of eHealth tools among vulnerable groups, although endogeneity is clearly a potential cause for concern.

**Table 4:** Results from meta-regression with adherence as an effect size measure.

Independent variables	Beta coefficient	SE	*P* value
Patient portal technology (yes=1, no=0)	1.37	0.73	.07
Mobile app technology (yes=1, no=0)	1.75	0.75	.13
Exclusive tool (yes=1, no=1)	.51	0.44	.25
Multimodal content (yes=1, no=0)	2.49^a^	0.72	.00
Training for using eHealth tool (yes=1, no=0)	−.51	0.56	.38
Interaction with health providers (yes=1, no=0)	1.23^a^	0.55	.03
Quality score of included study (minimum=0, maximum=31)	.49	0.78	.53
Constant	−3.72^b^	1.86	.06
Adjusted *R*^2^	38.80	—^c^	—
Τ^2^	1.086	—	—
I^2^	99.84	—	—

^a^*P*≤.05.

^b^*P*≤.10.

^c^Not applicable.

### Publication Bias Test

**Figure 3:**
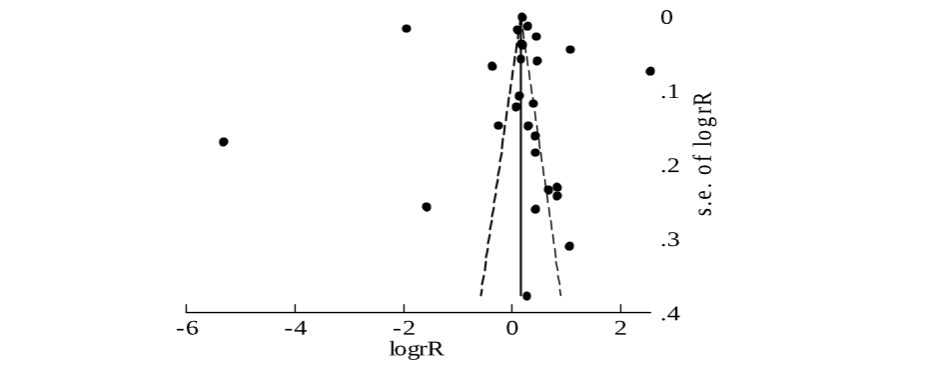
Funnel plot corresponding to Begg’s test (pseudo 95% confidence limits).

To estimate the between-study heterogeneity, we applied the Begg and Egger tests. The Begg test estimated the rank correlation between the effect size measure and its variance, and it is more appropriate because we used log (RR) as an estimate effect size measure. We also presented the graph for the Egger test because it is the most often reported test for publication biases [93]. Our results are presented in Table 5. The Egger test graph is shown in Figure 3.

**Figure 4:**
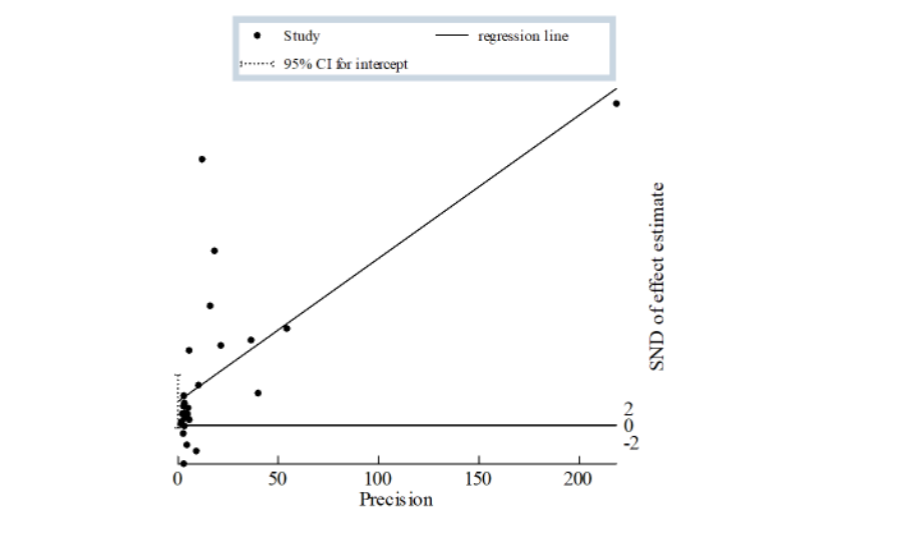
Regression line related to the Egger test.

The Begg test showed that there was no rank correlation between effect size measure and its variance. This means that there was no evidence of publication bias for this effect size. The results from the Egger test were consistent with that of the Begg test. The regression line shows that their publication bias does not seem to be present here (Figure 4).

**Table 5:** Results from the Begg correlation test.

Begg correlation test	Value
Adjusted Kendall score (P-Q)	−29
Standard deviation of score	47.97
Number of studies	27
z score	−0.60
Pr>|z|	0.545

## Discussion

### Principal Findings

Our first goal was to identify the level of adherence related to eHealth tools among vulnerable groups. As the adherence of eHealth tools is a precursor for their effectiveness, we hope that our results can help to identify the potentially effective tools for vulnerable groups. In this study, we compared the proportion of people who showed intended adherence with those who did not use eHealth tools. Our results show that the pooled level of intended adherence toward eHealth tools is 7% (95% CI −0.23 to 0.38), which implies that some people from vulnerable population groups used eHealth tools over time. However, the very small percentage (7%) implies that the number of adherers can be improved. This is consistent with the findings from previous studies [95]. They reported that the use of the internet is generally lower among vulnerable groups [94]. The small percentage (7%) in this study can be related to the high level of heterogeneity. In this review, we included studies with different designs (eg, longitudinal and RCT). This, among other factors, led to heterogeneity in the estimation of adherence levels. Furthermore, the difference in adherence levels can be observed among different vulnerable groups. In the United States, migrants show higher adherence levels than people from low-income groups or older adults when they use eHealth tools [40,53]. In this study, we included not only studies that involved different vulnerable groups but also those that addressed different health outcomes. This can also be an explanation for high heterogeneity.

Our second goal was to identify the design and implementation characteristics that influence the level of adherence within vulnerable groups. The results from the meta-regression show that design characteristics of eHealth tools, multimodal content and possibility of having a direct contact with the provider, are predictors of a higher adherence level. These two characteristics are assumed to mitigate the problems of health literacy and the digital divide among vulnerable groups. These results are particularly observed among eHealth tools that target minorities—one example is an eHealth tool for increasing knowledge on diabetes among African Americans [40]. The presence of multimodal content could increase the intrinsic motivation of participants and enable them to understand basic messages without language barriers. Furthermore, the use of multimodal content exceeds borders: eHealth tools are not only storage rooms for health information but also tools to *learn how to do things* or how to change health behavior. Direct interaction with providers without actual visits can save time. This is particularly important for single parents or people with low income and several jobs [40,83].

The low adherence among vulnerable groups and the fact that some design characteristics can improve adherence might imply that people from vulnerable groups will adhere to eHealth tools more if these tools are designed in accordance with their needs. For example, people diagnosed with high blood pressure might adhere more to Web-based portals if the portal shows a video on how to change your lifestyle instead of posting a text about healthy diets [28,96]. This is related not only to language barriers but also to the comprehension of health information. Joint dysfunctionality is another potential issue with low adherence. Joint dysfunctionality occurs when eHealth tools do not connect all health services. For example, participants may use both patient portals to refill their medications and Web-based tools to decrease their weight. However, these two tools and their data may not necessarily be connected. In case they are not connected, it may negatively affect adherence for both tools; the inclusion of both tools in daily routine may be perceived as too burdensome. Adherence is one of the precursors for effectiveness of eHealth tools. Our results suggest that, although small, adherence among vulnerable groups does exist, but it develops over time. This implies that eHealth tools do have the potential to decrease disparities among vulnerable groups.

The results from the systematic review also show that some users, although registered, never use eHealth tools. This can be explained by the fact that users might be registered by their provider. For example, GPs in The Bronx (the United States) usually register their patients to a patient portal during the regular appointment [33]. However, the registered patients never use patient portals or Web-based platforms. In other words, participants interested in eHealth tools register and continue as active users. Those without an interest in eHealth tools might be registered but without continuous use. This way eHealth tools attract a specific share of users among the vulnerable groups, and these users are consistently using the app. However, this creates the problem of how to attract new users within this population. Recent studies show that participants from vulnerable groups use eHealth tools less than other population groups [4]. One way to overcome this problem is to use *inclusive* tools that cover different population groups. This includes tools that are used by both younger and older users or by people from different social statuses. Another way is to capitalize on social ties and networks to expand the number of users [72]. For example, some eHealth tools allow for the use of encrypted chat groups for family members or for people with the same ethnical background.

Furthermore, our results show that design characteristics such as the type of technology (Web-based platform, mobile phones, or patient portals) have different patterns to address vulnerable groups. The most common types of technology used for health purposes are patient portals and Web-based platforms. They are different in design and purpose. Patient portals are characterized by direct interaction between the patient and the provider. They focus mostly on older adults or the chronically sick. Kp.org, a patient portal from the United States, is such an example. They also provide training for their users. For example, patients in nursing homes receive training for computer use and navigation through the portal [66]. This way, patient portals try to overcome problems associated with the digital divide. Conversely, Web-based platforms are usually *drivers* for tools that were developed before as *paper-and-pencil* version for general population groups [97]. Furthermore, most Web-based platforms are related to obesity. This is also related to the fact that the United States has the highest rates of obesity in the world and that most of our studies come from the United States [98]. Most Web-based platforms have a clear theoretical background and a clear evaluation plan. Web-based platforms usually benefit from multimodal content—they use videos or games to improve the adherence of their users [84]. This way they also overcome the problems of health literacy. They usually focus on one specific problem—obesity or diabetes—without connecting it to other aspects of patients’ health status. They are not always directly connected to other electronical data within the health care system. Conversely, patient portals are embodied within health care systems, but they also do not cover all aspects of health care. Usually, patient portals are developed for certain health care providers (certain hospitals or insurance companies). One of the examples is a patient portal for veterans in the US army known as My HealtheVet. This portal was created to address the special needs of veterans, and it is adjusted for specialized providers. The information from this portal is not connected with health care services outside of veterans’ clinics. It is also difficult to generalize the experience from this portal to that of similar eHealth tools [13]. If patient portals were linked to all providers and allowed patients to store information from different types of services, adherence to them might improve. For example, they do not always include prevention measures or possible therapeutic advice [41]. This can be important to improve effectiveness.

Our results also show that Web-based platforms are usually developed as *exclusive* tools for vulnerable groups—for example, for the gay population or Hispanic minorities [17,99,100]. This can be double sided as these groups might feel stigmatized in comparison with the general population with similar problems. Mobile phones are favored among certain vulnerable groups such as minorities that are trendsetters in their use [17]. However, our results show that only a small number of mobile health apps have been evaluated. One of the reasons might be that mobile phone apps are usually produced by small entrepreneurs. Their distribution does not require legal or ethical approval. In addition, they are very often not directly connected to health care systems [82].

### Limitations

The results of the meta-analysis related to the adherence of eHealth tools show a high level of heterogeneity. This was expected as we included different vulnerable groups, different eHealth tools, and different diseases that these tools address. Furthermore, we included studies with different designs such as RCTs, cohort studies, or observational studies. It would be useful to run meta-analyses related to adherence for each of the designs or for each of the vulnerable groups. Heterogeneity in our meta-analysis can be due to some eHealth tools being specifically related to certain health care institutions and that they cannot be applied in other institutions. This is important for the adherence rate—people who move from one nursing home to another cannot use the same eHealth tool anymore. Patient portals related to specific nursing homes are exemplary for this situation [77,78]. In addition, results based on users from one institution are difficult to extrapolate to the population level. This is emphasized by the lack of clear patterns for evaluating eHealth tools or deciding on outcome measures related to their effectiveness [9].

In this study, we also used meta-regression. We are aware that the number of included studies is small (N=27). This decreases the power of our analysis and might lead to biases. Furthermore, endogeneity is an issue.

Despite our efforts to perform all subsequent steps in the searching process carefully, we might have missed some relevant studies. This might be because of our definition of vulnerable groups and the ambiguities in the terminology of eHealth. In addition, the small number of included studies did not allow us to identify design and implementation characteristics per vulnerable group. In other words, we could not determine which design characteristics suits which group the best.

As we focused only on studies that have a reported adherence rate, this means that we excluded studies that evaluated eHealth tools using different measures. For example, some studies from low- and middle-income countries evaluate eHealth tools using only health outcomes or subjective measures such as quality of life or user satisfaction [50].

Although most European countries and the United States do have legal regulations about the use of eHealth tools, there is still a concern about the data collected via eHealth tools. In this study, we did not pay attention to legal and ethical considerations related to eHealth tools. This can be an interesting avenue for future research.

In the United States, many eHealth tools are funded through the federal government [54]. For example, the US government aims to spend US $38 billion in 10 years to develop eHealth for making health care more accessible. However, many end users (patients or medical providers) also pay for eHealth tools. Furthermore, many of the tools are funded by small entrepreneurs. In this study, we did not examine the source of funding and mechanisms of financing. Future research might benefit from including these characteristics.

### Conclusions

In conclusion, the use of eHealth among vulnerable population groups is still minimal. One way to improve adherence among vulnerable groups is to design eHealth tools with multimodal content. In addition, enabling direct communication between users and medical providers can improve access to health information among vulnerable groups. Future research should focus on evaluation studies on eHealth tools and health outcomes related to them, in addition to user satisfaction. Furthermore, future research should pay attention to defining intended adherence for different vulnerable groups and related eHealth tools. Providing eHealth tools that connect different health services would potentially improve the use not only among vulnerable groups but also in the general population. Although previous studies have emphasized that eHealth tools can be used to replace regular services, this can only be possible if eHealth tools are actively used.

To the best of our knowledge, this is the first study to synthesize the influence of design and implementation characteristics on adherence. Our results show that multimodal content—video and games—can be an incentive for use among vulnerable groups. In addition, direct communication with health care providers may increase adherence. However, the evidence is preliminary as it is based on cross-sectional analysis. These results are useful for the design of future eHealth tools. In this study, we assessed the level of intended adherence. However, we did not assess the effectiveness of eHealth tools. In other words, we did not assess the extent to which eHealth tools help vulnerable groups improve their health outcomes. Future research should also focus on the effectiveness of eHealth tools among vulnerable groups.

## Appendix

Multimedia Appendix 1 Preferred Reporting Items for Systematic Reviews and Meta-Analyses Checklist.

Multimedia Appendix 2 The characteristics of studies included in meta-analysis.

## Abbreviations

eHealth: electronic health
GP: general practitioner
ICROMS: Integrated quality Criteria for the Review of Multiple Study designs
PRISMA: Preferred Reporting Items for Systematic Reviews and Meta-Analysis
RCT: randomized controlled trial
RR: risk ratio
WHO: World Health Organization
