# Supplementary material for: Adherence to Electronic Health Tools Among Vulnerable Groups: Systematic Literature Review and Meta-Analysis
Source: J Med Internet Res. 2020 Feb 6;22(2):e11613. doi: 10.2196/11613 (PMC7055852; doi:10.2196/11613)
Supplement: Multimedia Appendix 1 [file jmir_v22i2e11613_app1.docx]

Appendix 1 – PRISMA Checklist

**Table 1 PRISMA Checklist (based on Liberati et al., 2009). Note: some checks are not applicable as they are meant for a meta-analysis, not a systematic review.**

| **TITLE** | | | page |
| --- | --- | --- | --- |
| Title | 1 | Identify the report as a systematic review, meta-analysis, or both. | 1 |
| **ABSTRACT** | | |  |
| Structured summary | 2 | Provide a structured summary including, as applicable: background; objectives; data sources; study eligibility criteria, participants, and tools; study appraisal and synthesis methods; results; limitations; conclusions and implications of key findings; systematic review registration number. | 2 |
| **INTRODUCTION** | | |  |
| Rationale | 3 | Describe the rationale for the review in the context of what is already known. | 3-4 |
| Objectives | 4 | Provide an explicit statement of questions being addressed with reference to participants, tools, comparisons, outcomes, and study design (PICOS). | 3-4 |
| **METHODS** | | |  |
| Protocol and registration | 5 | Indicate if a review protocol exists, if and where it can be accessed (e.g., Web address), and, if available, provide registration information including registration number. | N.A. |
| Eligibility criteria | 6 | Specify study characteristics (e.g., PICOS, length of follow-up) and report characteristics (e.g., years considered, language, publication status) used as criteria for eligibility, giving rationale. | 11-12 |
| Information sources | 7 | Describe all information sources (e.g., databases with dates of coverage, contact with study authors to identify additional studies) in the search and date last searched. | 9-11 |
| Search | 8 | Present full electronic search strategy for at least one database, including any limits used, such that it could be repeated. | 9 |
| Study selection | 9 | State the process for selecting studies (i.e., screening, eligibility, included in systematic review, and, if applicable, included in the meta-analysis). | 9-11 |
| Data collection process | 10 | Describe method of data extraction from reports (e.g., piloted forms, independently, in duplicate) and any processes for obtaining and confirming data from investigators. | 9-11 |

SEARCH STRATEGY:

*PubMed: ((((((((((e-Health[Title/Abstract] OR eHealth[Title/Abstract]) OR (("health"[MeSH Terms] OR "health"[All Fields]) AND ("Information (Basel)"[Journal] OR "information"[All Fields]) AND tecnologies[Title/Abstract])) OR patient portals[Title/Abstract]) OR telemedicine[Title/Abstract]) OR "social media"[MeSH Terms]) OR Facebook[Title/Abstract]) OR Twitter[Title/Abstract]) OR Web 2.0[Title/Abstract]) OR "internet"[MeSH Terms]) AND (("health"[MeSH Terms] OR "health"[All Fields]) AND disparities**[All Fields])) OR vulnerable [All Fields] OR disadvantaged [All Fields] and migrants [MESH]OR immigrants [MESH] OR low income [Title/Abstract] OR older adults [Title/Abstract]))*

*Cinahl: ((MH (e-health) OR TI ((eHealth or eHealth or health information technologies)) OR AB (social media or internet or Facebook or Twitter or Web 2.0)) AND ((MH (migrants) OR (MH(immigrants) OR (MH (low income) OR (MH (older adults)).*
