# Supplementary material for: Adherence to Electronic Health Tools Among Vulnerable Groups: Systematic Literature Review and Meta-Analysis
Source: J Med Internet Res. 2020 Feb 6;22(2):e11613. doi: 10.2196/11613 (PMC7055852; doi:10.2196/11613)
Supplement: Multimedia Appendix 2 [file jmir_v22i2e11613_app2.docx]

Table 6. The characteristics of studies included in meta-analysis.

| Author, year | Year | Country | Study design | Sample size | Quality score based on: | Number of people uptake (n1) | Number of people adherence (n2) | Probability of one-time users (*P*1) | Probability of continuous users (*P*2) | Log(RR) | SE (logRR) |
| --- | --- | --- | --- | --- | --- | --- | --- | --- | --- | --- | --- |
| 1. Kim et al,  2009. [66] | 2009 | United States | Cohort, 33 months | N=70, initial platform users | 22 | 70 | 53 | .39 | .29 | 0.29 | 0.15 |
| 2. Sarkar et al, 2010. [67] | 2010 | United States | Cohort study with post hoc randomization | N=20,188 | 20 | 4311 | 3922 | .31 | .28 | 0.10 | 0.01 |
| 3. Kerr et al ,2010. [68] | 2010 | United Kingdom | Cohort studies | N=168 patients | 18 | 39 | 66 | .19 | .39 | -5.32 | 0.17 |
| 4. Ancker et al, 2011. [33] | 2011 | United States | Retrospective longitudinal study | N=74,368 | 21 | 7138 | 5791 | .01 | .07 | −1.94 | 0.17 |
| 5. Goel et al, 2011. [69] | 2011 | United States | Retrospective longitudinal study | N=7088 | 18 | 4891 | 3717 | .69 | .52 | 0.28 | 0.01 |
| 6. Ronda et al, 2013. [35] | 2013 | The Netherlands | Cross-sectional survey was conducted and then patients were randomly chosen: n=1500 from the login group and n=3000 from nonlogin group | A survey in a sample of 12,793 diabetes patients | 21 | 758 | 632 | .17 | .14 | 0.19 | 0.04 |
| 7. Osborn et al, 2013. [70] | 2013 | United States | — | 75 | 18 | 13 | 62 | .17 | .83 | −1.57 | 0.26 |
| 8 Cullen et al, 2017. [71] | 2017 | United States | — | N=151 | 31 | 92 | 61 | .61 | .41 | 0.39 | 0.12 |
| 9. Joseph et al, 2013. | 2013 | United States | — | African American women, N=54 | 22 | 34 | 15 | .62 | .27 | 0.83 | 0.24 |
| 10. Joseph et al, 2016. | 2016 | United States | RCT^a^ | African American women aged 17-22 years, N=58 | 21 | 25 | 16 | .43 | .28 | 0.43 | 0.26 |
| 11. Campbell et al, 2015. | 2015 | United States | Pre- to posttreatment within the same group | American native people from Alaska with diagnosed alcohol problems n=68 | 21 | 40 | 26 | .58 | .38 | 0.42 | 0.18 |
| 12. Herring et al, 2014. | 2014 | United States | RCT | N=18 | 22 | 9 | 7 | .50 | .38 | 0.27 | 0.37 |
| 13. Billings et al, 2015. | 2015 | United States | RCT | N=83 | 23 | 45 | 39 | .54 | .46 | 0.16 | 0.15 |
| 14. Smith et al, 2015. | 2015 | United States | Cohort study, randomly selected older adults from cohort | N=538 | 22 | 206 | 287 | .32 | .46 | −0.36 | 0.07 |
| 15. Levy et al, 2015. | 2015 | United States | RCT | N=61 | 20 | 27 | 22 | .36 | .44 | −0.24 | 0.22 |
| 16. Jhamb et al, 2015. | 2015 | — | Retrospective cohort of outpatients | N=2803 | 26 | 1705 | 1098 | .61 | .39 | 0.45 | 0.03 |
| 17. Gordon & Hornbrook, 2016. | 2016 | United States | Data were automatically recorded when patient logged in, cohort kp.org | N=231,082 | 18 | 178171 | 149929 | .77 | .64 | 0.18 | 0.01 |
| 18. Nazi et al, 2013. | 2013 | United States | Cohort | N=681 | 20 | 390 | 246 | .57 | .36 | 0.46 | 0.06 |
| 19. Foster et al, 2015. [3] | 2015 | United States | Pregnancy (pre) versus postpartum (post) design with convenient sample | N=15 | 17 | 14 | 13 | .93 | .86 | 0.07 | 0.12 |
| 20. Ernsting et al, 2017. | 2017 | Germany | Population-based survey, cross-sectional design | N=4144 | 22 | 1600 | 521 | .38 | .13 | 1.07 | 0.04 |
| 21. Arcury et al, 2017. | 2017 | United States | Observational study | N=628 | 18 | 200 | 41 | .32 | .21 | 0.42 | 0.16 |
| 22. Aalbers et al, 2016. | 2016 | The Netherlands | Quasi-experimental prospective study with a pre- to postdesign | N=2972 | 18 | 2305 | 171 | .77 | .06 | 2.55 | 0.07 |
| 23. Cavallo et al, 2016. | 2016 | United States | Single-group pretest posttest design—5 months follow-up | 170 low-income women in reproductive age | 20 | 40 | 12 | .23 | .08 | 1.05 | 0.44 |
| 24. Steinberg et al, 2014. | 2014 | United States | RCT | N=194 | 20 | 97 | 86 | .5 | .44 | 0.13 | 0.11 |
| 25. Bickmore et al, 2016. | 2016 | United States | RCT | N=89 | 23 | 43 | 19 | .48 | .21 | 0.83 | 0.23 |
| 26. Ryan et al, 2013. | 2013 | United States | RCT | N=24 | — | 21 | 11 | .88 | .45 | 0.67 | 0.23 |
| 27. Buis et al, 2017. | 2017 | United States | RCT | N=123 | — | 63 | 53 | .51 | .43 | 0.17 | 0.14 |

Table 7. The design and implementation characteristics of studies included in meta-analysis.

| **Author, year** | Target group | Possibility of training | Exclusive/inclusive | Possibility of having direct contact with provider | Type of technology | Multimodal content |
| --- | --- | --- | --- | --- | --- | --- |
| 1. Kim et al,  2009. [66] | Older adults with low income (100% below US poverty line) who lived in nursing homes | 2 days per week they could receive assistance how to use the portal remark | Exclusive, the intervention was not specially designed for the target population. There was no language tailoring for immigrants. There are no multimodal contents such as graphics or videos. All possibilities are text based | Patients could share their records with providers or family members | Electronic Web-based health record that could be accessed via desk PCs | No just text-based co tent |
| 2. Sarkar et al, 2010. [67] | English-speaking patients diagnosed with diabetes (including minorities | Patient should show that they do have skills to use the internet | Exclusive in terms that it is developed for diabetes patients; it also includes information how to get insurance with this particular diagnosis | There is an interaction via sending an email to provider and making a Web-based appointment | Web-based portal known as Kp.org | No multimodal content |
| 3. Kerr et al ,2010. [68] | People diagnosed with cardiovascular diseases | Training for using the Web-based platform is provided | Inclusive intervention designed to include patients with different characteristics. It can be also applied to other medical conditions | Indirect interaction by asking questions | Web-based intervention | Multimodal content was not provided |
| 4. Ancker et al, 2011. [33] | Low-income population from New York area | No | Inclusive | Yes | Web-based secure portal known as MyChart active since 2008 in this area | No |
| 5. Goel et al, 2011. [69] | Racial/ethnical minorities | There was no training, but patient should show that they know how to use the portal | Inclusive | Interaction with physician was also available | Web-based secured patient portal MyChart | No only text |
| 6. Ronda et al, 2013. [35] | People diagnosed with diabetes type 1 or 2 | No | Exclusive, specially designed by Diamuraal and is copyrighted by Portavita B.V. (Amsterdam, The Netherlands) | Patients can seek contact with physicians after they enter their glucose values | Web-based secured portal | No, only text |
| 7. Osborn et al, 2013. [70] | Chronic ill patients | No | Inclusive for all users of primary health care | Yes | Web-based secured portal known as My Health At Vanderbilt | No, just text |
| 8 Cullen et al, 2017. [71] | Minorities | No | Exclusive | Yes | Web-based platform | Yes |
| 9. Joseph et al, 2013. | Minorities | Yes | Inclusive | Yes | Website | Yes |
| 10. Joseph et al, 2016. | African American women | No | Exclusive | — | Web-based intervention promoting physical activity among African American women. There is also an iPhone app available | Yes, videos, blogs, and possibility to share photos. |
| 11. Campbell et al, 2015. | Native Alaska Americans aged 18-30 years | No | Inclusive, made for general population | No | Web-based intervention to reduce drug abuse behavior | Yes, videos, texts role-model playing on the Web |
| 12. Herring et al, 2014. | Low-income and minority women after delivery | No | Exclusive, made for target group | Yes | Combination of Facebook and mobile phone intervention | Only text |
| 13. Billings et al, 2015. | African American women | No | Inclusive | No | Web-based intervention to promote use of condoms | Yes |
| 14. Smith et al, 2015. | Older adults aged 55-70 years | No | Inclusive | Yes | Patient portals | No only text |
| 15. Levy et al, 2015. | Low-income people diagnosed with diabetes, only those with unsatisfied glucose level | No | Exclusive, only low-income people with troubles in controlling their level of glucose | Yes | SMS and mobile calls. Providers can SMS on the Web-based platform | No, text and phone call |
| 16. Jhamb et al, 2015. | People with nephrology diseases | No | Inclusive | Yes | Individualized medical record | No just text |
| 17. Gordon & Hornbrook, 2016. | Older adults aged 65-79 years | Yes | Inclusive for all population groups | Yes | Patient portal known as kp.org. Also measures patient opinions about portal | No |
| 18. Nazi et al, 2013. | Veterans in US army | No | Exclusive | Yes | Patient portal | No just texts |
| 19. Foster et al, 2015. [3] | 16-31-year-old African American women | No | Exclusive for this group | Yes | Mobile app | No just texts |
| 20. Ernsting et al, 2017. | Low-income people | No | Inclusive | No | Mobile app | Yes |
| 21. Arcury et al, 2017. | African, Spanish low-income minorities | No | Inclusive | Yes | Patient portal | No, text |
| 22. Aalbers et al, 2016. | Older adults | No | Exclusive for target group | Yes | Web-based intervention | Yes, video games, graphs |
| 23. Cavallo et al, 2016. | Low-income women in reproductive age | No | Inclusive—made initially for general population and then modified | No | Social media group Web based | Yes |
| 24. Steinberg et al, 2014. | African American women | No | Exclusive | Yes | Web-based intervention combined with voice call | Yes |
| 25. Bickmore et al, 2016.[ | People with low health literacy | Yes | Exclusive for people with low health literacy | Yes | Web-based searching engine | Yes |
| 26. Ryan et al, 2013. | African Americans with diabetes | No | Exclusive | — | Web-based portal | No texts |
| 27. Buis et al, 2017. | African Americans with hypertension | No | Exclusive, designed for this group | — | SMS—mobile messages | No text only |
